# Encapsulation of Fennel and Basil Essential Oils in β-Cyclodextrin for Novel Biopesticide Formulation

**DOI:** 10.3390/biom14030353

**Published:** 2024-03-14

**Authors:** Nina Devrnja, Boban Anđelković, Jovana Ljujić, Tatjana Ćosić, Sofija Stupar, Milica Milutinović, Jelena Savić

**Affiliations:** 1 Institute for Biological Research “Siniša Stanković”—National Institute of the Republic of Serbia, University of Belgrade, Bulevar despota Stefana 142, 11108 Belgrade, Serbia; tatjana@ibiss.bg.ac.rs (T.Ć.); sofija.stupar@ibiss.bg.ac.rs (S.S.); milica.milutinovic@ibiss.bg.ac.rs (M.M.); savic.jelena@ibiss.bg.ac.rs (J.S.); 2 Faculty of Chemistry, University of Belgrade, Studentski trg 16, 11000 Belgrade, Serbia; aboban@chem.bg.ac.rs (B.A.); jovanalj@chem.bg.ac.rs (J.L.)

**Keywords:** inclusion complex, FT-IR, SPME/GC-MS, Colorado potato beetle

## Abstract

β-cyclodextrin (β-CD) is a good host for the encapsulation of fennel and basil essential oils (FEO and BEO, respectively) and the formation of inclusion complexes (ICs) using the co-precipitation method. According to the results of the GC/MS analysis conducted in this study, monoterpenes and monoterpenoids were the dominant chemical groups in total FEO, while in BEO, these two groups occurred along with sesquiterpenes and sesquiterpenoids. The presence of dominant compounds from both EOs was validated using the FT-IR spectra of ICs, which indicated successful complexation. Analyses conducted using SPME/GC-MS showed the continuous emission of volatiles over 24 h from both ICs. Under SEM, particles of both ICs appeared to have a rectangular or rhomboid morphology and few aggregates. The insecticidal properties of EOs and ICs with β-CD were tested on the Colorado potato beetle (CPB) as a model pest. The inclusion complex of β-CD with FEO altered the developmental dynamic and body mass of the CPB. The initial increase in the proteolytic activity of CPB larvae fed with potato plants sprayed with ICs was not maintained for long, and the proteolytic efficacy of treated larvae remained in line with that of the control larvae. Future investigations will focus on manipulating the volume of EOs used and the treatment duration for optimal efficacy and potential application.

## 1. Introduction

Science represents a link between education, society, and economy that can reduce environmental degradation, social tensions, and inequalities and improve human quality of life, as well as enabling sustainable life cycles. Decreasing the use of and finding alternatives to detrimental chemical pesticides are among the major aims of the scientific community, in line with the initiatives of the diverse Sustainable Development Goals (SDGs) adopted by all United Nations Member States, which support the global transition to healthy and sustainable agri-food systems. Although modern agriculture depends heavily on pesticides, boosting yields and profit must be counterbalanced with irreversible damage to nature, and the European Union’s (EU’s) Farm to Fork Strategy within the Green Deal includes an initiative to reduce pesticide use and associated risks by 50% by 2030 [1]. However, when we change our focus from strategies and documents toward reality, the EU is the largest pesticide export market in the world [2] and exports pesticides that are banned by its own member states due to the weak legal restrictions of other countries. Incongruously, the EU depends heavily on agricultural imports, and a huge portion of these imports come from countries to which the largest EU pesticide companies export banned pesticides. The net result is that neither the public nor the environment experience any benefit. Despite all of the SDGs and “green” strategies and initiatives, traces of pesticides from agriculture can still be detected in food and feed, soil, and water ecosystems, resulting in a negative impact on the health and fitness of humans, domestic animals, and non-target beneficial organisms. The current literature reports elevated rates of chronic diseases among people exposed to pesticides [3,4], which affect not only target organisms but the whole environment, remaining there from a few hours to several years, and can be transported long distances from areas of application [5]. The long-term effects of pesticides can result in outcomes ranging from changes of habitats and the food chain to large-scale negative effects on biodiversity [6,7,8].

Undoubtedly, the research initiatives to find pesticide alternatives should be established and intensified, aiming to meet and uphold all of the objectives from extant legal policies and trajectories. It is essential that we learn from nature and work with it, not against it. Within this context, biopesticide research, as a part of integrated pest management, may provide beneficial data to enable a shift away from pesticide dependency and contribute to sustainable agriculture. Biopesticides are defined as chemicals with pesticide activity that are derived from natural sources, such as plants or microorganisms, and can be used for pest management [9]. Plants respond to biotic or abiotic stresses through a complex network of secondary metabolites, with many exhibiting pesticide activities. These metabolites, accumulated in different parts of the plant, are components of plant extracts or essential oils (EOs) and frequently manifest toxicity, act as antifeedants through pest metabolism alternation and fitness reduction, or simply deter or repel insect pests [10,11,12]. EOs, as complex mixtures of volatile compounds, have been shown to display insecticidal properties against various pests [13,14,15]. They are already recognized as potential biopesticides involved in sustainable agriculture [16,17,18,19,20,21,22], but with challenging requirements to adapt results from laboratories to fluctuating outdoor conditions.

The challenges that need to be overcome regarding EO-based biopesticides are their volatility, limited persistence under environmental conditions, and water insolubility [23]. Light, temperature, and oxygen can accelerate autoxidation processes and limit EO activity [24,25,26]. Encapsulation is a method that has been developed to overcome these limitations to the use of EOs as biopesticides. In light of EO encapsulation, cyclodextrins (CDs), a group of cyclic oligosaccharides obtained from starch, have earned a significant reputation primarily as excipients for the solubilization and stabilization of poorly soluble drug candidates in aqueous solutions. Progress in biotechnology from the 1970s enabled the massive production of CDs, leading to their successful industrial application. Nowadays, the industrial exploitation of CDs is far-reaching and is still increasing thanks to their highly adaptable properties. Currently, the global CD market is mainly segmented into the food, cosmetic, and pharmaceutical industries. They are used as food additives to eliminate undesirable tastes or odors and improve sensorial qualities, shelf life, and the sequestration of components [27]. In pharmacy, CDs increase the physicochemical stability of drugs, modify their delivery sites, and prolong their shelf life [28]. Investigations within green chemistry use CDs as multitask agents for metal nano-heterogeneous catalysis [29,30]. The CD market is continually expanding through the introduction of various derivatives for special industrial exploitation and is expected to undergo significant growth in the coming years [31].

**Figure 1:**
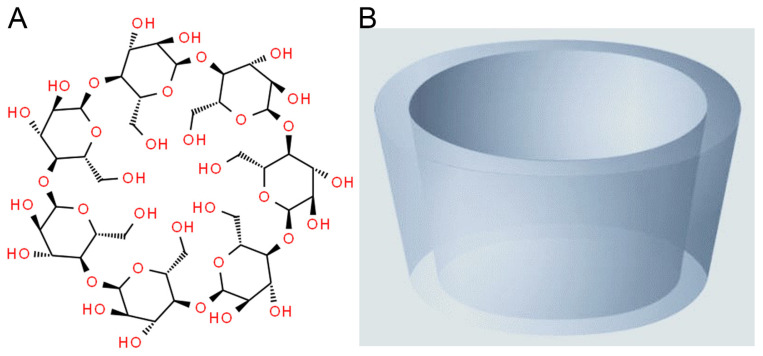
Chemical structure (**A**) and 3-D model (**B**) of β-cyclodextrin molecule.

The main types of natural CDs are α-CD, β-CD, and γ-CD, which contain six, seven, or eight glucopyranose units, respectively. Beta-cyclodextrins (β-CDs) are α-1,4-linked cyclic oligosaccharides with seven glucose units (Figure 1A). According to the Food and Drug Administration (FDA, USA), they are “generally recognized as safe” (GRAS). The partial-cone-shaped β-CD molecule (Figure 1B) has a hydrophilic exterior that confers solubility in water and a hydrophobic interior cavity that forms inclusion complexes with various hydrophobic compounds through hydrophobic interactions between cyclodextrin cavity walls and guest molecules [32,33]. The hydroxyl groups of cyclodextrin, primary and secondary, are positioned on the narrower and broader rims, respectively [34]. Primary hydroxyl groups can reduce the diameter of cyclodextrin’s cavity through rotation, while secondary groups form strong hydrogen bonds and contribute to the molecule’s rigidity [35]. These properties of β-CD ensure protection of the bioactivity of the encapsulated guest molecule. Many studies have been published reporting the complexation of β-CD with pure EOs or with certain EO components isolated for different purposes [36,37,38,39,40,41].

It was previously demonstrated that the EO obtained from basil (*Ocimum basilicum* L.) exhibits high efficacy against various insect pests [42,43,44], while fennel (*Foeniculum vulgare* Mill.), with its characteristic anise odor and its use in medicine and the food industry, was also found to have applications in plant protection [24,45,46,47]. As both EOs have properties that can be used for developing new formulations of botanical pesticides, we investigated the possible encapsulation of these oils with β-CD, as host molecules, and characterized the obtained inclusion complexes (ICs). Finally, we tested the efficacy of individual EOs and ICs against a major potato pest, the Colorado potato beetle (CPB; *Leptinotarsa decemlineata* Say), one of the world’s most destructive and globally invasive insect herbivores. The CPB is present in almost all areas with a suitable climate, except for some areas protected by large water barriers or with less optimal climatic conditions, like Scandinavia [48,49]. Since the CPB has an impressive feeding rate and high fecundity, potato farms might be destroyed if this fascinating insect is not managed. Thus, the hypothesis tested in the present work states that the prolonged evaporation of EO volatiles within ICs, and thus, the prolonged exposure of larvae, may have a stronger impact on CPB growth and development.

## 2. Materials and Methods

### 2.1. Isolation of Essential Oils

The plant material of fennel (*Foeniculum vulgare* Mill.) and basil (*Ocimum basillicum* L.) used for EO isolations was collected at the blooming stage from a private garden near Belgrade (Šimanovci, Belgrade nearby, Serbia) and dried in the shade at room temperature. Essential oils were obtained from the dried herbs using Clevenger-type apparatus during 3 h of hydrodistillation. The isolated EOs from fennel (FEO) and basil (BEO) were kept in dark glass bottles at a temperature of 4 °C until use.

### 2.2. Identification of EO Constituents Using Gas Chromatography with a Flame Ionization Detector (GC/FID) and Gas Chromatography/Mass Spectrometry (GC/MS)

The essential oils were diluted in dichloromethane (Sigma-Aldrich, Wien, Austria, ≥99.9%, capillary GC grade) at a concentration of 10 µL mL^−1^. An Agilent 7890A GC system equipped with a 5975C inert XL EI/CI MSD and an FID detector, connected by a capillary flow technology 2-way splitter with make-up gas, was used for gas chromatography (GC) and gas chromatography/mass spectrometry (GC/MS) analyses. A DB-5 MSI capillary column (30 m × 0.25 mm × 0.25 µm film thickness) was used. The column temperature was linearly programmed in a range of 60–240 °C at a rate of 3 °C min^–1^. Helium (He) was the carrier gas, with a flow rate of 1.0 mL min^–1^ at 210 °C (constant pressure mode). The samples were injected in split mode (10:1). The injection volume was 1 µL, and the injector temperature was 220 °C. The transfer line was heated to 240 °C, and the detector temperature was 300 °C. EI mass spectra (70 eV) were obtained in the *m*/*z* range of 35–550 atomic mass units (amu). The ion source and quadrupole temperatures were 300 °C and 150 °C, respectively.

A library search and mass spectral deconvolution and extraction were performed using NIST AMDIS (Automated Mass Spectral Deconvolution and Identification System) software, version 2.64.113.71, using retention index (RI) calibration data analysis parameters with a “strong” level and a 10% penalty for compounds without an RI. The retention indices were experimentally determined using a standard method involving the retention times of n-alkanes, injected after the EOs under the same chromatographic conditions.

### 2.3. Formation of Beta-Cyclodextrin Essential Oil (β-CD-EO) Inclusion Complexes (ICs)

Inclusion complexes of the selected EOs in β-CD were prepared using a modified co-precipitation method [50,51]. Briefly, 9 g of β-CD (white powder, percent purity—98%; water-soluble; product of Acros Organics) was diluted in 25% ethanol (10 mL) and placed on a magnetic stirrer at a constant temperature (50 °C) to obtain a saturated solution. Next, 10 mL of the ethanolic solution of each EO (10%) was added drop by drop into β-CD and placed on a magnetic stirrer at a constant temperature (50 °C) until dry to allow the formation of ICs. The dry mix of EO and β-CD was stirred constantly until it was cold, and then left at −20 °C overnight. The following day, the mix was lyophilized for 24 h, and then condensed in a condenser at 50 °C. The white powders obtained were stored in a sealed container inside a desiccator until use.

### 2.4. Characterization of ICs

#### 2.4.1. Fourier Transform Infrared Spectroscopy (FT-IR)

Infrared spectra of the β-CD-EO inclusion complexes (β-CD-FEO and β-CD-BEO), each EO (FEO and BEO), and β-CD alone were measured on a Nicolet summit FT-IR spectrometer (Thermo Fisher, Waltham, MA, USA). Samples were measured using attenuated total reflection mode (ATR) on diamond crystal with 32 scans for the samples and background and a resolution of 4 cm^−1^ in the range of 4000 to 400 cm^−1^. Omnic Paradigm 1.0 software was used for measurement, and Omnic 7.0 software for spectra processing. All spectra were baseline, ATR-corrected, and normalized to the largest pic.

#### 2.4.2. Headspace Solid-Phase Microextraction with Gas Chromatography and Mass Spectrometry (SPME/GC-MS) Quantification of EO Volatiles over Time

To measure the quantity of EO release over time, headspace solid-phase microextraction with gas chromatography and mass spectrometry (SPME-GC/MS) analysis was performed. The volatile release was quantified in triplicate using approximately 5.5 mg of IC samples and 5 µL of the internal standard methyl undecanoate at a concentration of 0.01 ppm (1 nL mL^−1^). The volatile release was measured after 0, 3, 6, and 24 h of exposure at room temperature and 20% relative humidity. Samples were measured in a 20 mL headspace vial; then, the standard solution was added, and the vial was immediately sealed with polytetrafluoroethylene (PTFE) thread tape and capped. The SPME analysis was performed using a manual SPME arrow injection kit with polydimethylsiloxane (PDMS), a 95 µm thickness, and a fiber measuring 10 mm in length. The SPME fiber was inserted into the vial and exposed to the volatile components contained in the IC samples, which were allowed to fill the headspace, during an incubation period of 20 min at 35 °C in a water bath. Desorption was performed in the GC inlet for 20 s, with samples additionally “flushed” for 300 s in the continuum to be reconditioned. Blank samples were run to validate the reconditioning process and quantification method. The same GC program was used for identifying the constituents of the EOs with a split mode of 10:1.

#### 2.4.3. Scanning Electron Microscopy (SEM)

Scanning electron microscopy (SEM) was used for investigating the morphology and approximate size of the crystals formed within the ICs. Leaves of potato plants were sprayed with β-CD-FEO and β-CD-BEO ICs that had previously been dissolved in water (10 µL EOs per treatment). For SEM, sections of the sprayed potato leaves were coated with a thin layer of gold in a BAL-TEC SCD 005 sputter coater (BAL-TEC GmbH, Schalksmühle, Germany). The samples were then observed using a JEOL JSM-6390 LV scanning electron microscope (JEOL, Tokyo, Japan), and the properties of the crystals were determined. All samples were observed at a 20 kV electron velocity. The dimensions of the crystals were determined using ISIS JEOLSEM software Smile View Ver. 2.26. Measurements from 10 crystals were taken for each β-CD-EO complex.

### 2.5. Bioassay with Colorado Potato Beetle (CPB)

#### 2.5.1. Experimental Setup

Potato (*Solanum tuberosum* L.) was used as a model plant for the insect bioassay. The potato plants (cultivar Désirée) were germinated from tubers collected from a chemically untreated field (Šimanovci, Belgrade nearby, Serbia) and maintained in individual glass jars as previously described [52]. After 18 days, fully expanded potato plants were exposed to evaporating EOs or sprayed with β-CD-FEO or β-CD-BEO. Essential oils (10 µL) were applied to Whatman 3 filter papers (1 cm × 1 cm), which were placed in the jars on metal holders, avoiding contact with the plant parts and allowing the EOs to evaporate. The inclusion complexes were previously dissolved in water so that the amount of EO was the same as in the jars with EO alone. The potato plants were exposed to EOs or sprayed with ICs on three consecutive days. In the jars containing control plants, no plants were sprayed with ICs, and no EO was added. Five jars with potato plants were used for each experimental group. The jars in all of the experimental groups were tightly closed with lids and sealed with parafilm for 8 h each day, and were then opened until the new application the next day. On the 4th day, CPB larvae (2nd instar) were distributed into the jars, where they stayed until they had completed their life cycles and adults emerged; the process is described below.

Leaves with CPB egg clusters were collected from pesticide-untreated field-grown potato plants during the summer of 2021 (Šimanovci, Belgrade nearby, Serbia). The leaves were placed in plastic Petri dishes (90 mm in diameter) on moist filter paper and incubated at 24 ± 1 °C. After the larvae started to hatch, fresh untreated potato leaves were added daily.

Second-instar larvae were randomly selected from different clusters, and 10 larvae (*n* = 50 larvae for each experimental group) were distributed into each jar onto potato plants that had previously been treated with EOs or ICs for three consecutive days. Larval body mass and mortality were recorded on the 4th and 8th day after the start of the experiment until the end of the CPB life cycle.

#### 2.5.2. Total Protease Activity of CPB

The activity of total digestive proteases was analyzed in 3rd- and 4th-instar larvae fed with potato plants in each experimental group. The extraction of digestive enzymes and a protease activity assay were performed as described in our previous report [53]. In brief, whole larvae were homogenized in 0.9% NaCl (1:5 *w*/*v*), and the total protease activity was determined using azocaseine as the substrate at pH 6.5 [54]. The protease activity was expressed in enzyme units (U) per mg of total proteins, where one enzyme unit represents the amount of enzyme required for an absorbance change of 1.0 during 1 h under the conditions of the assay.

#### 2.5.3. Statistical Analysis

The data from the larval mass and proteolytic activity measurements were subjected to one-way analysis of variance (ANOVA) using SAS software (SAS Institute, 2002. SAS/STAT, ver. 9.00. SAS Institute Inc., Cary, NC, USA), and the results for each time point are presented as means ± standard errors (SE) (*n* = 10). The means were separated using the LSD test at *p* ≤ 0.05.

## 3. Results

### 3.1. Identification of EO Constituents Using GC/FID and GC/MS

**Figure 2:**
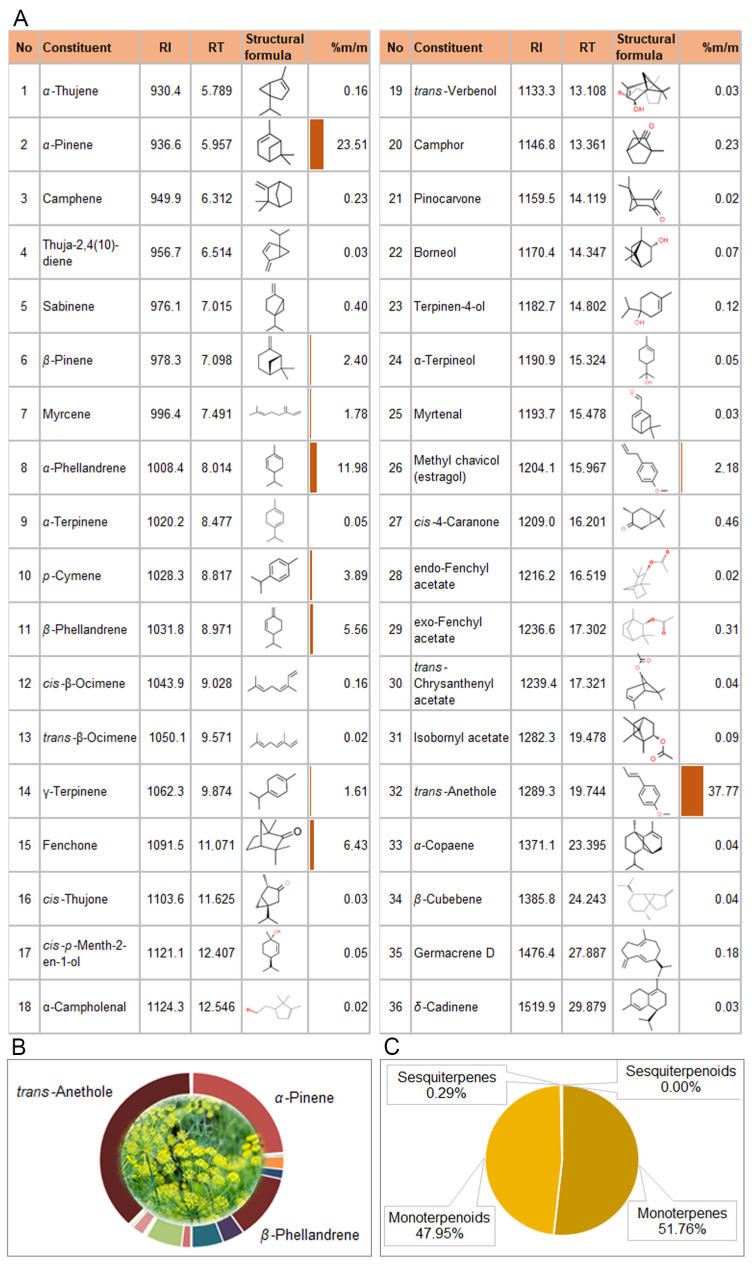
Chemical composition (**A**); dominant constituents (**B**); and chemical groups (**C**) of fennel (*Foeniculum vulgare* Mill.) essential oil. RI—retention index; RT—retention time; %m/m—% of compound in total oil. Colored bars represent the contribution (%m/m) of the compound in total EO.

**Figure 3:**
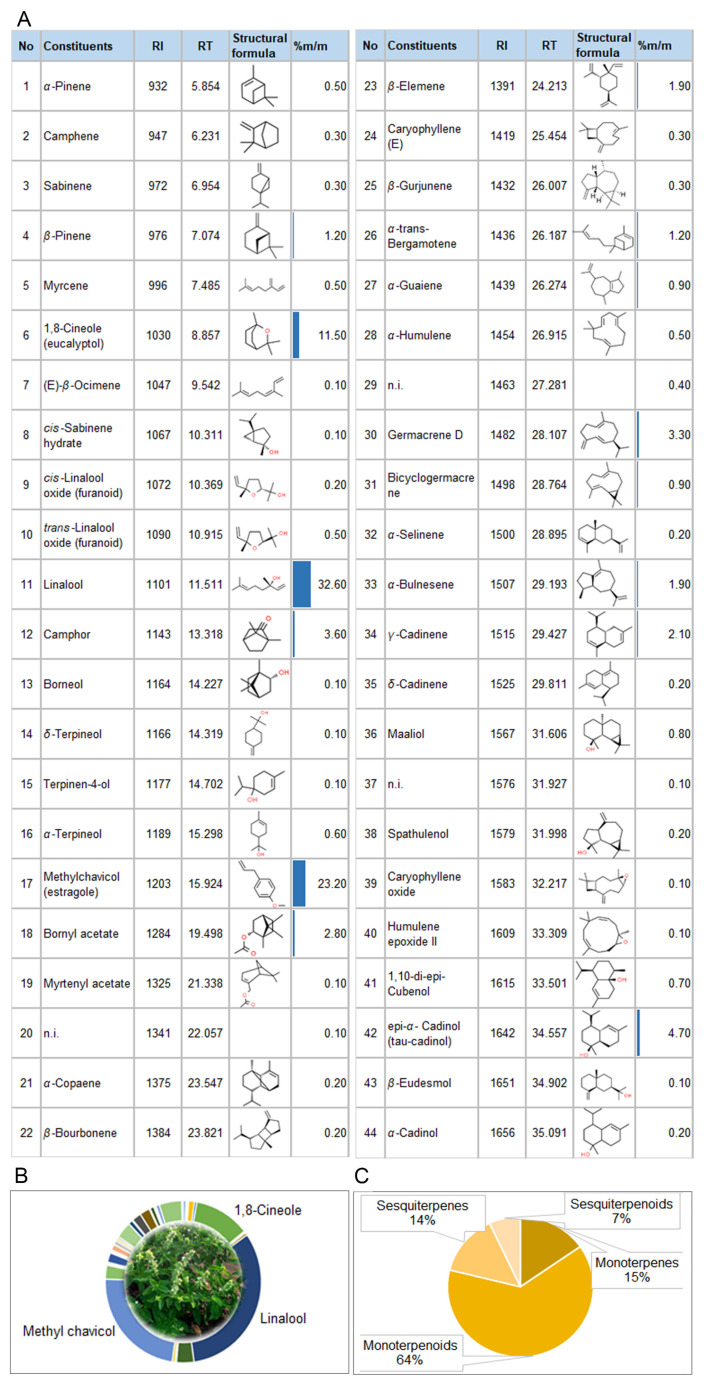
Chemical composition (**A**); dominant constituents (**B**); and chemical groups (**C**) of basil (*Ocimum basilicum* L.) essential oil. RI—retention index; RT—retention time; n.i.—non-identified; %m/m—% of the compound in total oil. Colored bars represent the contribution (%m/m) of the compound in total EO.

Gas chromatography was coupled with mass spectrometry for the quantitative analysis of fennel (Figure S1) and basil EOs (Figure S2). The volatile compounds detected are listed in Figure 2 and Figure 3. A total of 36 compounds from fennel essential oil (FEO) were detected and identified, representing 100% of the total oil (Figure 2A). This EO was characterized by a high content of the trans-anethole (37.77%), followed by α-pinene (23.51%) and α-phellandrene (11.98%) (Figure 2B).

The majority of volatiles from FEO were assigned to monoterpenes (51.76%) and monoterpenoids (47.95%), while volatiles from the sesquiterpenes class were present in a small percentage (0.29%) in the total oil (Figure 2C).

According to the results of the GC/MS analysis, 41 of the 44 volatile compounds detected in basil EO (BEO) were identified, which represents 99.4% of the total oil (Figure 3A). Linalool (32.6%) and methyl chavicol (estragole) (23.2%) were the dominant constituents of this EO, followed by 1,8-cineol (12%) (Figure 3B). Monoterpenoids were the dominant class of volatiles (63.2%) in BEO, accompanied by monoterpenes (15.2%), sesquiterpenes (14.1%), and sesquiterpenoid compounds (6.9%) (Figure 3C).

### 3.2. FT-IR Validation of IC Formation

**Figure 4:**
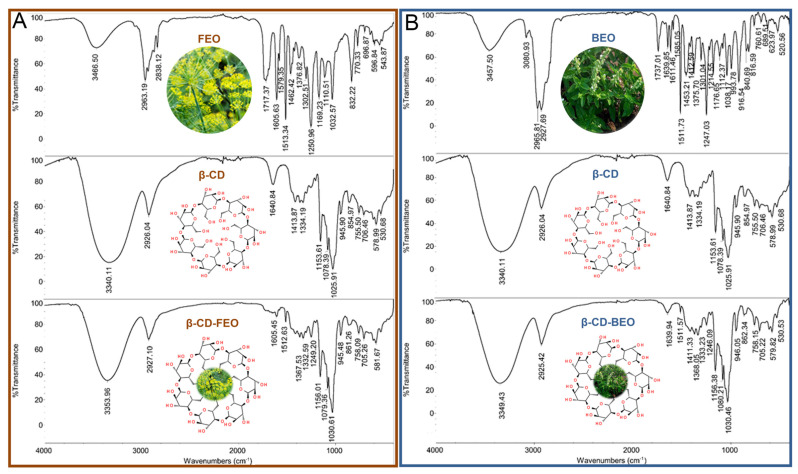
FT-IR spectra of fennel EO (FEO), β-cyclodextrin (β-CD), and IC of β-cyclodextrin and fennel EO (β-CD-FEO) (**A**) and FT-IR spectra of basil EO (BEO), β-cyclodextrin (β-CD), and IC of β-cyclodextrin and basil EO (β-CD-BEO) (**B**).

FT-IR spectra, used for the confirmation of IC formation, were obtained from each EO (FEO and BEO), the ICs (β-CD-FEO and β-CD-FEO), and β-CD alone, and are presented in Figure 4A,B. In the FT-IR spectra of the EOs, a wide strip of O–H valence vibrations above 3000 cm^−1^ sp^2^ was present, along with C–H streaking vibrations above 3000 cm^−1^ sp^3^. In the range of 1740 to 1000 cm^−1^, strips from C=O, C=C, and C–O stretching vibrations were detected. Other skeleton bending vibrations, such as the umbrella of CH_3_ vibrations =C–H, C–H, C–O C=C, C–C, etc., were present in the range of 1450 to 400 cm^−1^. The vibration strips present in β-CD were typical for a cyclic glucose oligomer with a dominant valence of O–H and C–O vibrations.

**Figure 5:**
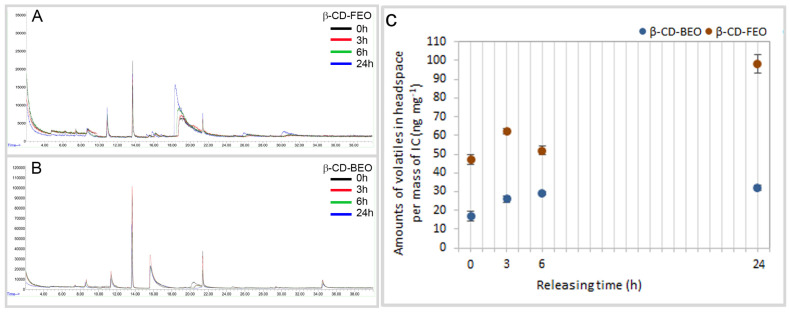
SPME/GC-MS chromatograms of β-CD-FEO (**A**), β-CD-BEO (**B**), and volatile release from ICs after 0, 3, 6, and 24 h (n = 3, *p* < 0.05) (**C**). At 13.71 and 21.43 min, there are cyclomethylsiloxane signals from the SPME GC/MS system.

According to the results of the GC/MS analysis, *p*-cymene, fenchone, methyl chavicol, and trans-anethole represent almost 50% of FEO (Figure 2A), which can also be seen in the FT-IR spectrum (Figure 4A) as well as in the SPME/GC-MS chromatograms of β-CD-FEO (Figure 5A), and some of their characteristic strips are present in the FT-IR spectrum of β-CD-BEO (Figure 4B). The stretching of C=C skeleton vibrations at 1511 cm^−1^ and vibration at 1245 cm^−1^ from C–O stretching appear under the β-CD signals. In the IR spectrum of the presented complex, the shifting of skeleton strips of CD toward higher wavenumbers, from 1365.0, 1153.6, 1078.4, 1025.9, and 755.5 cm^−1^ to 1368, 1156, 1079, 1031, and 758 cm^−1^, respectively, indicates a strong physical interaction and successful complexation [55,56]. A similar case was present in the β-CD-BEO complex (Figure 4B and Figure 5B). In BEO, linalool and methyl chavicol were dominant, with a total prevalence of 55% (Figure 3A). All other vibrations of EO constituents present in the ICs overlapped with similar vibrations of β-CD.

### 3.3. Volatile Release over Time

The release of EO volatiles from ICs was quantified after 0, 3, 6, and 24 h using SPME/GC-MS (Figure 5). At the start (0 h), β-CD-FEO emitted more than twice the amount of volatiles (47 ± 2 ng mg^−1^) emitted from β-CD-BEO (17 ± 2 ng mg^−1^) (Figure 5C). The investigated complexes showed the continuous emission of volatiles from the IC produced from FEO and less consistent emission over time in the case of the IC produced from BEO. Overall, fennel volatiles were emitted on a much larger scale, up to 100 ± 9 ng mg^−1^ in comparison to 32 ± 5 ng mg^−1^ from the IC of basil, after 24 h.

### 3.4. SEM Validation of Obtained ICs

**Figure 6:**
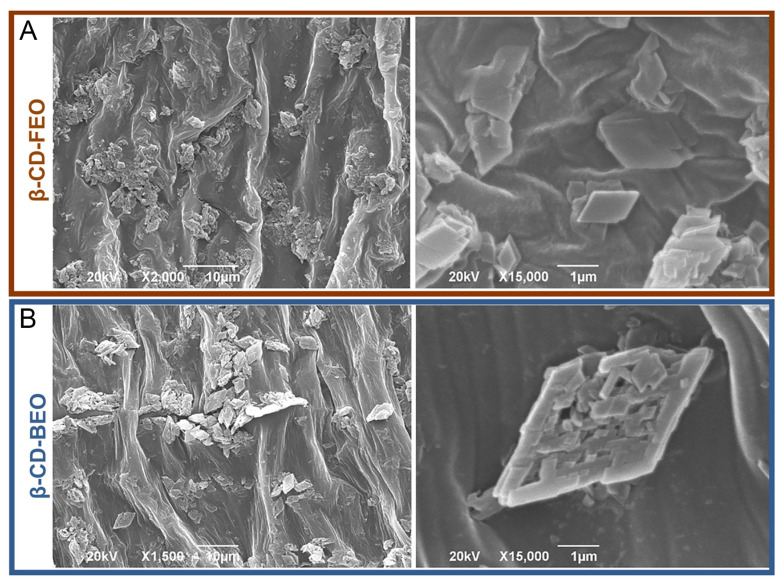
Scanning electron micrographs of β-CD-FEO at 2000× and 15,000× (**A**); scanning electron micrographs of β-CD-BEO at 1500× and 15,000× (**B**).

SEM analysis confirmed the formation of both ICs. The morphologies of the ICs of β-CD-FEO and β-CD-BEO are shown in Figure 6A,B. Particles of both ICs appeared to have a rectangular or rhomboid lamellate morphology, although the IC particles of β-CD-BEO appeared more elongated. It was noted that particles of both ICs self-associated and formed aggregates. The average size of β-CD-FEO particles was around 828 nm, while particles of β-CD-BEO ranged around 1115 nm on average.

### 3.5. Bioassay with Colorado Potato Beetle

**Figure 7:**
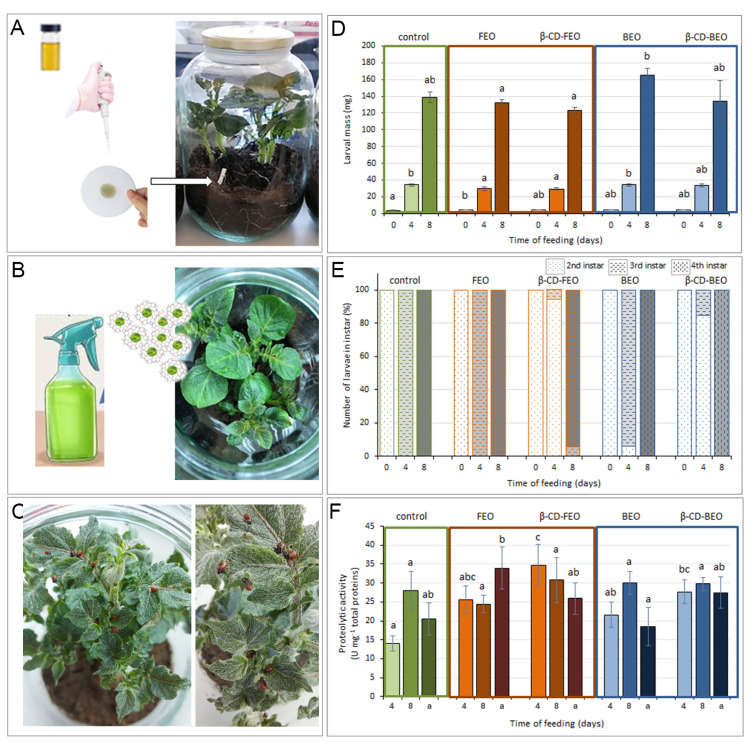
Effect of fennel (FEO) and basil (BEO) essential oils and β-CD inclusion complexes (β-CD-FEO and β-CD-BEO) on growth, development, and proteolysis of Colorado potato beetle (CPB) larvae. Application of EOs (**A**), spraying with ICs (**B**), and feeding of CPB larvae with EOs or ICs on treated or non-treated (control) potato plants (**C**). Larval mass (**D**), percentage of larvae in 2nd, 3rd, and 4th instars (**E**), and proteolytic activity of larvae and adults (a) (**F**) are presented before treatment (day 0) and after larvae were fed with potato plants for 4 and 8 days. Results in (**D**,**F**) are presented as means ± standard errors (n = 10). Means denoted by the same letter within the same time point are not significantly different (*p* ≤ 0.05) according to the LSD test.

Feeding on potato plants previously exposed to fennel or basil EO (Figure 7A) or sprayed with individual ICs (β-CD-FEO or β-CD-BEO) (Figure 7B) altered the growth, dynamics of development, and proteolytic activity of CPB larvae (Figure 7C).

On day 4 of the experiment, larvae fed with potato plants sprayed with β-CD-FEO, as well as larvae fed with potato plants that were previously exposed to FEO alone, had reduced weight (25 ± 1 mg and 25 ± 2 mg) compared to the control larvae (30 ± 1 mg) and larvae fed with potato plants exposed to BEO or sprayed with β-CD-BEO (30 ± 1 mg and 30 ± 2 mg) (Figure 7D). On the same day, it was also noted that all larvae (100%) from the control and fennel EO groups had molted to the third instar, while larvae from the BEO group (6%) and both IC experimental groups (94.5% for β-CD-FEO and 84.72% β-CD-BEO) were still in the second instar. This retardation in developmental dynamics remained until the 8th day of the experiment in larvae fed with potato plants sprayed with β-CD-FEO. A total of 5.71% of larvae from this experimental group were still in the third instar, while larvae from all other experimental groups had already molted to the fourth instar (Figure 7E). On the 8th day, larvae fed with potato plants sprayed with β-CD-FEO, as well as larvae fed with potato plants that were previously exposed to FEO, had reduced mass (119 ± 4 mg and 128 ± 3 mg, respectively) compared to the control larvae (135 ± 6 mg). In contrast, larvae fed with potato plants exposed to BEO had increased mass (161 ± 9 mg) compared to the control. Additionally, no significant mortality of larvae was noticed in any experimental group.

Feeding on treated potato plants affected the proteolytic activity of the tested larvae. On day 4 of the experiment, larvae fed with potato plants sprayed with β-CD-FEO had significantly increased proteolytic activity (35 ± 6 U mg^−1^ of total proteins) compared to the control larvae (14 ± 2 U mg^−1^ of total proteins). This trend was also noticed in larvae fed with potato plants sprayed with β-CD-BEO (28 ± 3 U mg^−1^ of total proteins) and those exposed to fennel EO (26 ± 4 U mg^−1^ of total proteins) and basil EO (22 ± 3 U mg^−1^ of total proteins). However, on day 8 of the experiment, larvae from all experimental groups exhibited proteolytic activity in line with the control larvae (Figure 7F).

## 4. Discussion

The essential oils (EOs) used in this study were obtained from aromatic plants belonging to the Apiaceae (fennel) and Lamiaceae (basil) families. Taxonomic distance is reflected in the chemical composition of these two oils. Fennel EO (FEO) was rich in monoterpenes and their derivatives (99.71%), with high amounts of trans-anethol and α-pinene in the total oil. Although monoterpenoids were also the major chemical group (63.2%) in basil EO (BEO) that contained linalool, a naturally occurring terpene alcohol, and methyl chavicol, a phenolic derivative, as dominant compounds, this oil was characterized by a significant amount of sesquiterpenes and their derivatives (21%). The ultimate performance of the final products containing EOs is impacted by the fact that many of these compounds are easily oxidized or destroyed in the presence of oxygen, light, or heat. The use of protective complexing agents such as cyclodextrins (CDs) has increased in recent decades to improve, preserve, and enable the controlled release of unstable bioactive compounds. The current literature suggests that β-CD has better complexation efficiency than α-CD and γ-CD, and it might complex between 80 and 99% of volatile compounds [57]. However, the presence of different chemical components can influence inclusion complex (IC) formation with β-CD. The chemical structure and properties of β-CD enable it to encapsulate guest molecules and carry aromatic substances using different preparation methods, leading to the formation of ICs without the formation or tearing of covalent bonds [38,40,58,59]. Most volatile molecules have a molecular weight between 120 and 160 gmol^−1^, allowing β-CD molecules to entrap them and act as a reservoir of volatile components [60]. However, the majority of sesquiterpenes have a molecular weight above 200 gmol^−1^, which makes monoterpenes the main candidates for inclusion in the cavity of β-CD molecules. Thus, trans-anethole, with a molecular weight of 148 gmol^−1^, or α-pinene, with a molecular weight of 136 gmol^−1^, as dominant volatiles in FEO, are perfect for nesting into the cavity of β-CD. Similar chemical features can be seen in linalool (154 gmol^−1^) and methyl chavicol (148 gmol^−1^) from basil EO. These features make volatiles from FEO and BEO ideal candidates for encapsulation with β-CD.

The next step in determining the efficacy of EO complexation is the choice of preparation technique. Various preparation techniques result in the formation of ICs with different features, performance, and volatile bioavailability. For obtaining ICs in a solid state, the co-precipitation method and the kneading method are commonly used [61]. The modified co-precipitation method used in our study was found to be effective for the formation of ICs of β-CD with FEO and BEO, and included the use of ethanol as a co-solvent for both EOs and β-CD. The greater proportion of water (75%) compared to ethanol (25%) affects the flexibility of the β-CD molecule and simplifies the formation of ICs and volatile nesting, while the addition of ethanol to water may improve the solubility of β-CD [62]. The co-precipitation technique was used for the successful IC formation of β-CD with linalool [58] and trans-anethole [63]. The findings of previous studies that have examined the complexation between β-CD and trans-anethole indicated that the encapsulation of trans-anethole occurred through either its methoxy or ethylene group penetrating the CD cavity [57,64]. The FT-IR spectrum of β-CD-FEO in our study indicated high relative concentrations of trans-anethole and α-pinene in the obtained IC.

The IC of BEO with β-CD, obtained using the crystallization method, was found to contain a higher relative concentration of the main compounds (linalool and methyl chavicol) in the EO recovered from the complex than in the pure EO [65]. These authors concluded that the enhanced relative concentration of linalool and methyl chavicol in the IC was primarily due to their high concentrations in the pure EO, which enabled better molecular encapsulation during the complexation process. The same volatiles are dominant in the BEO investigated in our study, and the FT-IR spectra indicated that the relative concentration of these compounds was also high in the formed IC. The results of the study by Hădărugă et al. [65] also showed that terpene hydrocarbons are better encapsulated with β-CD than their corresponding oxygenated ones. Although linalool and methyl chavicol are oxygenated compounds, they are excellent candidates for encapsulation, especially the highly flexible linalool, due to their rigid and hydrophobic structure and small dimensions.

It is important in the assessment of IC formation to include various analytical methods and experimental techniques based on any variation in a physical or chemical feature of the guest molecule [66,67], and the results must be combined, connected, and compared to reach a valid conclusion. The most common techniques for the characterization of IC include FT-IR and SEM. FT-IR is the main technique used to determine the presence of functional groups. FT-IR analysis was found to be convenient in detecting the interaction between CDs and EOs [68,69,70,71]. The successful formation of ICs in our study was verified based on variations in the position, shape, and intensity of the peaks on the FT-IR spectra. The appearance of new resultant peaks indicated bond formation between the host and guests. FT-IR is a cheap, fast, simple, and reliable technique able to confirm the presence of formed complexes.

The quality of the obtained ICs was additionally sustained using SEM. The recorded micrographs showed tiny aggregates of particles with lamellate morphology, with no fractures on the surface texture. The detected aggregates can be classified by size as stable microparticles that can precipitate from aqueous media but disassemble upon medium dilution [72]. The particles were similar to the IC of β-CD containing curcumin, lemongrass oil, geranium oil, eucalyptus oil, clove oil, and oregano oil [73,74,75].

In our study, SPME/GC-MS was used to detect and quantify concentrations of volatiles released over time from each inclusion complex into the headspace. Altogether, the release pattern of the IC with FEO differed from that with BEO. β-CD-FEO released a greater amount of volatiles at the start (0 h). Both ICs exhibited a more-or-less continuous release of volatiles over time. However, fennel volatiles discharged from the IC, after the initial increase, declined after 3 h, and thereafter exhibited a continual increase by the 24th hour. This outcome shows promise regarding the desired longevity of volatiles. According to the literature [76], methods for the preparation of ICs affect volatiles released over time; thus, it can be concluded that the modified co-precipitation method used is adequate for the formation of stable ICs with β-CD and fennel or basil EOs. This method was found to be cheap, simple, and suitable for non-water-soluble substances [33]. The conversion of liquid and volatile compounds into crystalline form can render such materials suitable for the manufacture of powders, granules, or tablets.

The continual increase in volatiles released within 24 h is significant for the potential application of these ICs as biopesticides. The encapsulation of EOs with β-CD may complement all of the features of EOs required to be considered a useful biological strategy to mitigate any pest. The obtained ICs were tested against the CPB, the most important insect defoliator of potato crops and currently one of the major pests in agronomy. It was estimated that about 40–50 cm^2^ of potato leaves are consumed by a single beetle during its larval stage [77]. This pest has remarkable adaptability to variable climates and environmental stresses and undergoes prompt evolutionary change, which strengthens its ability to rapidly evolve resistance to almost all registered insecticides or their active ingredients [78,79,80,81,82]. Considering that plants and pests co-evolve, host-plant resistance is one of the best solutions for controlling the CPB. Plant secondary metabolites represent an evolutionary response to different abiotic or biotic stresses, and EOs are mixtures of compounds included in plant chemical defense, which makes them promising candidates for use as biopesticides. The encapsulation of fennel and basil EOs, as oils with recognized insecticidal action, with β-CD enabled longer exposure of potato plants and target pests to volatiles.

The molecular targets of insecticide action are coupled with the nervous system, midgut and hormone activity, and molting of insects [83]. Although no mortality of CPB larvae was detected during our experiment, feeding on potato plants previously sprayed with individual ICs or exposed to fennel or basil EO for 8 h altered their larval growth, dynamics of development, and proteolytic activity. CPB larvae fed with potato plants sprayed with β-CD-FEO, as well as larvae fed with potato plants that were previously exposed to FEO, had reduced weight compared to the control larvae and larvae fed with potato plants exposed to BEO or sprayed with β-CD-BEO. It can be assumed that treatment with FEO and FEO IC resulted in reduced feeding in CPB larvae because the detection of taste compounds regulates feeding decisions, or because the conversion of food into body mass was less efficient, although the proteolytic activity of these larvae was high at the beginning. In a previous study, trans-anethole showed highly antifeedant activity against white moth (*Hyphantria cunea* Drury) larvae, in addition to the inhibition of digestion, accompanied by an imbalance in metabolic and oxidative processes [84]. Additionally, trans-anethole acted synergistically with some minor constituents of EOs in terms of the feeding deterrence of insects [85,86].

Using the Colorado potato beetle as a model, our study demonstrated the presence of a general response of these insects to host potato plants that were directly or indirectly exposed to EO volatiles. Because the supply of nitrogen is their primary limiting factor, insect herbivores must effectively digest plant proteins to survive. The CPB has serine and cysteine digestive proteases, but cysteine proteases contribute mostly to proteolytic activity in the gut [87,88]. A possible explanation for the initial elevated total proteolytic activity in all experimental groups compared to the control could be that the adaptive response to new volatiles in the potato plants’ atmosphere involves a general increase in defense-related compounds, which consequently affected the total gut proteolytic activity of CPB larvae [52]. Insect herbivores use olfaction, which utilizes numerous chemosensory gene families, to detect volatile organic molecules in order to locate their host plants in various landscapes. Although the CPB has adapted to feeding on potato plants, its sophisticated adaptations to overcome plant responses continue to evolve. Insects’ highly flexible midguts enable them to quickly adjust to changing nutritional levels in their food [89]. Rivard et al. [90] monitored digestive proteases in the CPB after feeding larvae with plants pre-treated with either methyl jasmonate or arachidonic acid, two compounds inducing different sets of defense genes in potato. Larvae fed with treated plants were negatively affected compared to larvae fed with non-treated plants, suggesting the capability of both compounds to induce partial resistance in potato plants toward the CPB. The authors gave a possible explanation for this phenomenon, suggesting that it was triggered by the accumulation of protein inhibitors in the potato plant following treatment. However, these larvae partially compensate for the presence of defense-related proteins by adjusting their digestive proteolytic system, both quantitatively and qualitatively, which may be a possible explanation for our results, since high proteolytic activity was detected after 4 days of feeding. The results of Stupar et al. [52] showed that there was an alternation in the content of soluble monosaccharides in potato plants that had been exposed to French marigold EO for 8 h, which was reflected in decreased levels of fructose and glucose, while the sucrose level was increased. It is well known that sucrose is among the major nutrients responsible for stimulating feeding behavior in CPB larvae [91]. However, our results indicated that after the initial increase, CPB larvae adapted their proteolytic machinery to the presence of modified nutrition patterns in leaf tissues. Thus, their total gut proteolytic activity remained in the same range as that of the control larvae, which suggests that this is one way to establish a successful feeding strategy for this pest. In the field, where plant growth is continuously hampered by natural enemies or unfavorable abiotic circumstances, it appears that CPBs may modify their digestive proteolytic metabolism in response to changes generated in their host by environmental/chemical cues.

Both applied ICs altered larval developmental dynamics; thus, the larvae fed with potato plants that were sprayed with ICs were late in molting from the second to the third instar relative to the control. This retardation in developmental dynamics remained until the 8th day of the experiment in larvae fed with potato plants sprayed with the IC with FEO. A previous study by Devrnja et al. [12] highlighted that tansy (*Tanacetum vulgare* L.) EO ingested by gipsy moth (*Lymantria dispar* L.) larvae caused molting delay. The EO appeared to be more efficient in delaying molting when incorporated into their food, which could be the case for molting delay in larvae fed with plants that were sprayed with ICs that potentiate the prolonged persistence of EO compounds on the potato leaf surface. Not reaching the critical mass for the induction of the molting process [92] could be a potential reason for such a result when taking into account the reduced mass of these larvae compared to other experimental groups. These observations are interesting in terms of the possible foliar applications of ICs with FEO for the induction of starvation stress in the target field population of CPB larvae. Starvation stress might be a good synergistic strategy for enhancing the upcoming application of biopesticides or other IPM measures against CPBs with altered immunity. Additionally, the increased duration of their instars makes CPB larvae more susceptible to pathogens, predators, and competitors [88,93].

Despite the numerous scientific reports on the promising effects of ICs with EOs on detrimental pests, no commercially available products intended for agronomical use have been registered. Following the development of stabile ICs and the proven toxic effect on the targeted organism, this approach should be more intensively tested against non-target beneficial organisms and under the pressure of environmental fluctuations.

## 5. Conclusions

This is the first report regarding the larvicidal/insecticidal activity of fennel and basil EOs encapsulated with β-CD against CPBs. The formation of an inclusion complex with β-CD ameliorated the volatility of fennel and basil EO constituents. There was a continuous emission of volatiles over time from β-CD-FEO and less consistent emission in the case of volatiles from the β-CD-BEO inclusion complex. Scanning electron micrographs showed that the particles of both ICs had a rectangular or rhomboid lamellate morphology with a larger average β-CD-FEO particle size.

Feeding CPBs with potato plants sprayed with ICs or exposed to individual EOs did not cause mortality in the larvae. However, it did alter their development; fennel EO and β-CD-FEO affected CPB larvae by reducing larval body mass and prolonging their development. Despite the initial increase in the proteolytic activity of CPB larvae on day 8 of the experiment, larvae from all experimental groups exhibited proteolytic activity in line with the control larvae.

This study presents the successful complexation of fennel and basil EOs with β-CD and hints at the insecticidal potential of the tested EOs and obtained ICs. Since the CPB is a pest with fascinating pesticide adaptability, every result that alters its fitness deserves additional investigation, contributing to cumulative global knowledge that will help develop successful management strategies. Future research projects will focus on manipulating the volume of EOs used and the treatment duration for optimal efficacy and potential field application.

## Data Availability

Data are contained within the article.

## Supplementary Materials

The following supporting information can be downloaded at: https://www.mdpi.com/article/10.3390/biom14030353/s1, Figure S1: Chromatogram of GC/MS analysis of fennel (*Foeniculum vulgare* Mill.) essential oil. Figure S2: Chromatogram of GC/MS analysis of basil (*Ocimum basilicum* L.) essential oil.

## References

[1] EC-European Commission 2020 (2020). A Farm to Fork Strategy for a Fair, Healthy and Environmentally-Friendly Food System.

[2] The Pesticide Atlas (2022). Facts and Figures about Toxic Chemicals in Agriculture.

[3] Mostafalou S., Abdollahi M. (2013). Pesticides and human chronic diseases: Evidences, mechanisms, and perspectives. Toxicol. Appl. Pharmacol..

[4] Arab A., Mostafalou S. (2022). Neurotoxicity of pesticides in the context of CNS chronic diseases. Int. J. Environ. Health Res..

[5] de Souza R.M., Seibert D., Quesada H.B., de Jesus Bassetti F., Fagundes-Klen M.R., Bergamasco R. (2020). Occurrence, impacts and general aspects of pesticides in surface water: A review. Process Saf. Environ. Prot..

[6] Geiger F., Bengtsson J., Berendse F., Weisser W.W., Emmerson M., Morales M.B., Ceryngier P., Liira J., Tscharntke T., Winqvist C. (2010). Persistent negative effects of pesticides on biodiversity and biological control potential on European farmland. Basic Appl. Ecol..

[7] Groh K., Vom Berg C., Schirmer K., Tlili A. (2022). Anthropogenic chemicals as underestimated drivers of biodiversity loss: Scientific and societal implications. Environ. Sci. Technol..

[8] Zaller J.G., Kruse-Plaß M., Schlechtriemen U., Gruber E., Peer M., Nadeem I., Formayer H., Hutter H.P., Landler L. (2022). Pesticides in ambient air, influenced by surrounding land use and weather, pose a potential threat to biodiversity and humans. Sci. Total Environ..

[9] Ayilara M.S., Adeleke B.S., Akinola S.A., Fayose C.A., Adeyemi U.T., Gbadegesin L.A., Omole R.K., Johnson R.M., Uthman Q.O., Babalola O.O. (2023). Biopesticides as a promising alternative to synthetic pesticides: A case for microbial pesticides, phytopesticides, and nanobiopesticides. Front. Microbiol..

[10] Obeng-Ofori D., Freeman F.D.K. (2001). Efficacy of products derived from *Ricinus communis* (L.) and *Solanum nigrum* (L.) against *Sitophilus oryzae* (L.) and *Tribolium castaneum* (Herbst) in stored maize. Ghana J. Agric. Sci..

[11] Nerio L.S., Olivero-Verbel J., Stashenko E. (2010). Repellent activity of essential oils: A review. Bioresour. Technol..

[12] Devrnja N., Kostić I., Lazarević J., Savić J., Ćalić D. (2020). Evaluation of tansy essential oil as a potential “green” alternative for gypsy moth control. Environ. Sci. Pollut. Res..

[13] Attia S., Mansour R., Abdennour N., Sahraoui H., Blel A., Rahmouni R., Lebdi Grissa K., Mazzeo G. (2022). Toxicity of *Mentha pulegium* essential oil and chemical pesticides toward citrus pest scale insects and the coccinellid predator *Cryptolaemus montrouzieri*. Int. J. Trop. Insect Sci..

[14] Giordani C., Spinozzi E., Baldassarri C., Ferrati M., Cappellacci L., Santibañez Nieto D., Pavela R., Ricciardi R., Benelli G., Petrelli R. (2022). Insecticidal Activity of Four Essential Oils Extracted from Chilean Patagonian Plants as Potential Organic Pesticides. Plants.

[15] Devrnja N., Gašić U., Šajkunić S., Cingel A., Stupar S., Tubić L., Savić J. (2022). UHPLC-OrbiTrap MS Characterization of Phenolic Profiles in French Marigold Extracts and Analysis of Their Antifeedant Activity against Colorado Potato Beetle. Plants.

[16] Isman M.B., Miresmailli S., Machial C. (2011). Commercial opportunities for pesticides based on plant essential oils in agriculture, industry and consumer products. Phytochem. Rev..

[17] Radwan E.M., El-Malla M.A., Fouda M.A., Mesbah R.A.S. (2018). Appraisal of Positive Pesticides Influence on pink bollworm larvae, *Pectinophora gossypiella* (Saunders). Egypt. Acad. J. Biol. Sci. F Toxicol. Pest Control.

[18] Ikbal C., Pavela R. (2019). Essential oils as active ingredients of botanical insecticides against aphids. J. Pest Sci..

[19] Isman M.B. (2020). Commercial development of plant essential oils and their constituents as active ingredients in bioinsecticides. Phytochem. Rev..

[20] Gonçalves A.L. (2021). The use of microalgae and cyanobacteriain the improvement of agricultural practices: A review on their biofertilising, biostimulating and biopesticide roles. Appl. Sci..

[21] Liu X., Cao A., Yan D., Ouyang C., Wang Q., Li Y. (2021). Overview of mechanisms and uses of biopesticides. Int. J. Pest Manag..

[22] Acheuk F., Basiouni S., Shehata A.A., Dick K., Hajri H., Lasram S., Yilmaz M., Emekci M., Tsiamis G., Spona-Friedl M. (2022). Status and Prospects of Botanical Biopesticides in Europe and Mediterranean Countries. Biomolecules.

[23] Turek C., Stintzing F.C. (2013). Stability of essential oils: A review. Compr. Rev. Food Sci. Food Saf..

[24] Isman M.B. (2000). Plant essential oils for pest and disease management. Crop Prot..

[25] Ložienė K., Venskutonis P.R. (2005). Influence of environmental and genetic factors on the stability of essential oil composition of *Thymus pulegioides*. Biochem. Syst. Ecol..

[26] Scott R.P.W., Worsfold P., Townshend A., Poole C. (2005). Essential oils. Encyclopedia of Analytical Science.

[27] Hu J., Du P., Liu S., Liu Q., Deng W. (2021). Comparative Study on the Effect of Two Drying Methods on the Guest Encapsulation Behavior of Osmanthus Flavor-2-Hydroxypropyl—Cyclodextrin Inclusion Complex. Flavour Fragr. J..

[28] Carneiro S.B., Duarte F.I.C., Heimfarth L., Quintans J.D.S.S., Quintans-Júnior L.J., Júnior V.F.D.V., De Lima A.A.N. (2019). Cyclodextrin-drug inclusion complexes: In vivo and in vitro approaches. Int. J. Mol. Sci..

[29] Noël S., L’eger B., Ponchel A., Sadjadi S., Monflier E. (2021). Cyclodextrins as multitask agents for metal nano-heterogeneous catalysis: A review. Environ. Chem. Lett..

[30] Fenyvesi É., Sohajda T. (2022). Cyclodextrin-enabled green environmental biotechnologies. Environ. Sci. Pollut. Res..

[31] Kfoury M., Fourmentin S. (2023). Cyclodextrins as building blocks for new materials. Beilstein J. Org. Chem..

[32] Duan X., Chen S., Chen J., Wu J. (2012). Enhancing the cyclodextrin production by synchronous utilization of isoamylase and α-CGTase. Appl. Microbiol. Biotechnol..

[33] Cheirsilp B., Rakmai J. (2016). Inclusion complex formation of cyclodextrin with its guest and their applications. Biol. Eng. Med..

[34] Franzini R., Ciogli A., Gasparrini F., Ismail O.H., Villani C. (2018). Recent developments in chiral separations by supercritical fluid chromatography. Chiral Analysis: Advances in Spectroscopy, Chromatography and Emerging Methods.

[35] Przybyla M.A., Yilmaz G., Remzi Becer C. (2020). Natural cyclodextrins and their derivatives for polymer synthesis. Polym. Chem..

[36] Wang J., Cao Y., Sun B., Wang C. (2011). Physicochemical and release characterisation of garlic oil-β-cyclodextrin inclusion complexes. Food Chem..

[37] Hill L.E., Gomes C., Taylor T.M. (2013). Characterization of beta-cyclodextrin inclusion complexes containing essential oils (trans-cinnamaldehyde, eugenol, cinnamon bark, and clove bud extracts) for antimicrobial delivery applications. LWT-Food Sci. Technol..

[38] Abarca R.L., Rodriguez F.J., Guarda A., Galotto M.J., Bruna J.E. (2016). Characterization of beta-cyclodextrin inclusion complexes containing an essential oil component. Food Chem..

[39] Wadhwa G., Kumar S., Chhabra L., Mahant S., Rao R. (2017). Essential oil–cyclodextrin complexes: An updated review. J. Incl. Phenom. Macrocycl. Chem..

[40] Cai L., Lim H., Nicholas D.D., Kim Y. (2020). Bio-based preservative using methyl-β-cyclodextrin-essential oil complexes for wood protection. Int. J. Biol. Macromol..

[41] Ma J., Fan J., Xia Y., Kou X., Ke Q., Zhao Y. (2023). Preparation of aromatic β-cyclodextrin nano/microcapsules and corresponding aromatic textiles: A review. Carbohydr. Polym..

[42] Kéita S.M., Vincent C., Schmit J.P., Arnason J.T., Bélanger A. (2001). Efficacy of essential oil of *Ocimum basilicum* L. and *O. gratissimum* L. applied as an insecticidal fumigant and powder to control *Callosobruchus maculatus* (Fab.) [Coleoptera: Bruchidae]. J. Stored Prod. Res..

[43] Govindarajan M., Sivakumar R., Rajeswary M., Yogalakshmi K. (2013). Chemical composition and larvicidal activity of essential oil from *Ocimum basilicum* (L.) against *Culex tritaeniorhynchus*, *Aedes albopictus* and *Anopheles subpictus* (Diptera: Culicidae). Exp. Parasitol..

[44] Rodríguez-González Á., Álvarez-García S., González-López Ó., Da Silva F., Casquero P.A. (2019). Insecticidal properties of *Ocimum basilicum* and *Cymbopogon winterianus* against Acanthoscelides obtectus, insect pest of the common bean (*Phaseolus vulgaris*, L.). Insects.

[45] Pavela R. (2018). Essential oils from *Foeniculum vulgare* Miller as a safe environmental insecticide against the aphid *Myzus persicae* Sulzer. Environ. Sci. Pollut. Res..

[46] Sayed Ahmad B., Talou T., Saad Z., Hijazi A., Cerny M., Kanaan H., Chokr A., Merah O. (2018). Fennel oil and by-products seed characterization and their potential applications. Ind. Crops Prod..

[47] Abdel-Baki A.A., Aboelhadid S.M., Sokmen A., Al-Quraishy S., Hassan A.O., Kamel A.A. (2021). Larvicidal and pupicidal activities of *Foeniculum vulgare* essential oil, trans-anethole and fenchone against house fly *Musca domestica* and their inhibitory effect on acetylcholinestrase. Entomol. Res..

[48] Weber D.C., Capinera J.L. (2008). Colorado potato beetle, *Leptinotarsa decemlineata* (Say) (Coleoptera: Chrysomelidae). Encyclopedia of Entomology.

[49] Lyytinen A., Boman S., Grapputo A., Lindström L., Mappes J. (2009). Cold tolerance during larval development: Effects on the thermal distribution limits of *Leptinotarsa decemlineata*. Entomol. Exp. Appl..

[50] Martins A.D., Craveiro A., Machado M., Raffin F., Moura T., Novák C., Éhen Z. (2007). Preparation and characterization of Mentha x villosa Hudson oil–β-cyclodextrin complex. J. Therm. Anal. Calorim..

[51] Seo E.J., Min S.G., Choi M.J. (2010). Release characteristics of freezedried eugenol encapsulated with β-cyclodextrin by molecular inclusion method. J. Microencapsul..

[52] Stupar S., Dragićević M., Tešević V., Stanković-Jeremić J., Maksimović V., Ćosić T., Devrnja N., Tubić L., Cingel A., Vinterhalter B. (2021). Transcriptome profiling of the potato exposed to french marigold essential oil with a special emphasis on leaf starch metabolism and defense against Colorado potato beetle. Plants.

[53] Devrnja N., Milutinović M., Savić J. (2022). When scent becomes a weapon—Plant essential oils as potent bioinsecticides. Sustainability.

[54] Michaud D., Nguyen-Quoc B., Yelle S. (1994). Production of Oryzacystatins I and II in *Escherichia coli* using the glutathione S-transferase gene fusion system. Biotechnol. Prog..

[55] Wang Y., Jiang Z.T., Li R. (2009). Complexation and molecular microcapsules of *Litsea cubeba* essential oil with β-cyclodextrin and its derivatives. Eur. Food Res. Technol..

[56] Jiang Z.T., Tan J., Tan J., Li R. (2016). Chemical components and molecular microcapsules of Folium *Artemisia argyi* essential oil with β-cyclodextrin derivatives. J. Essent. Oil-Bear. Plants.

[57] Ciobanu A., Landy D., Fourmentin S. (2013). Complexation efficiency of cyclodextrins for volatile flavor compounds. Food Res. Int..

[58] Al-Nasiri G., Cran M.J., Smallridge A.J., Bigger S.W. (2018). Optimisation of β-cyclodextrin inclusion complexes with natural antimicrobial agents: Thymol, carvacrol and linalool. J. Microencapsul..

[59] Xiao Z., Zhang Y., Niu Y., Ke Q., Kou X. (2012). Cyclodextrins as carriers for volatile aroma compounds: A review. Carbohydr. Polym..

[60] Das S., Gazdag Z., Szente L., Meggyes M., Horváth G., Lemli B., Kunsági-Máté S., Kuzma M., Kőszegi T. (2019). Antioxidant and antimicrobial properties of randomly methylated β cyclodextrin–captured essential oils. Food Chem..

[61] Paiva-Santos A.C., Ferreira L., Peixoto D., Silva F., Soares M.J., Zeinali M., Zafar H., Mascarenhas-Melo F., Raza F., Mazzola P.G. (2022). Cyclodextrins as an encapsulation molecular strategy for volatile organic compounds–pharmaceutical applications. Colloids Surf. B Biointerfaces.

[62] Bouchemela H., Madi F., Nouar L. (2019). DFT investigation of host–guest interactions between α-Terpineol and β-cyclodextrin. J. Incl. Phenom. Macrocycl. Chem..

[63] Zhang W., Li X., Yu T., Yuan L., Rao G., Li D., Mu C. (2015). Preparation, physicochemical characterization and release behavior of the inclusion complex of trans-anethole and β-cyclodextrin. Food Res. Int..

[64] Kfoury M., Auezova L., Greige-Gerges H., Ruellan S., Fourmentin S. (2014). Cyclodextrin, an efficient tool for trans-anethole encapsulation: Chromatographic, spectroscopic, thermal and structural studies. Food Chem..

[65] Hădărugă D.I., Hădărugă N.G., Costescu C.I., David I., Gruia A.T. (2014). Thermal and oxidative stability of the *Ocimum basilicum* L. essential oil/β-cyclodextrin supramolecular system. Beilstein J. Org. Chem..

[66] Mura P. (2014). Analytical techniques for characterization of cyclodextrin complexes in aqueous solution: A review. J. Pharm. Biomed. Anal..

[67] Mura P. (2015). Analytical techniques for characterization of cyclodextrin complexes in the solid state: A review. J. Pharm. Biomed. Anal..

[68] Kotronia M., Kavetsou E., Loupassaki S., Kikionis S., Vouyiouka S., Detsi A. (2017). Encapsulation of oregano (*Origanum onites* L.) essential oil in β-cyclodextrin (β-CD): Synthesis and characterization of the inclusion complexes. Bioengineering.

[69] Marques C.S., Carvalho S.G., Bertoli L.D., Villanova J.C., Pinheiro P.F., Dos Santos D.C., Yoshida M.I., de Freitas J.C., Cipriano D.F., Bernardes P.C. (2019). β-Cyclodextrin inclusion complexes with essential oils: Obtention, characterization, antimicrobial activity and potential application for food preservative sachets. Food Res. Int..

[70] Yuan C., Wang Y., Liu Y., Cui B. (2019). Physicochemical characterization and antibacterial activity assessment of lavender essential oil encapsulated in hydroxypropyl-beta-cyclodextrin. Ind. Crops Prod..

[71] Tian Y., Yuan C., Cui B., Lu L., Zhao M., Liu P., Wu Z., Li J. (2022). Pickering emulsions stabilized by β-cyclodextrin and cinnamaldehyde essential oil/β-cyclodextrin composite: A comparison study. Food Chem..

[72] Loftsson T., Sigurdsson H.H., Jansook P. (2023). Anomalous Properties of Cyclodextrins and Their Complexes in Aqueous Solutions. Materials.

[73] Rachmawati H., Edityaningrum C.A., Mauludin R. (2013). Molecular Inclusion Complex of Curcumin–β-Cyclodextrin Nanoparticle to Enhance Curcumin Skin Permeability from Hydrophilic Matrix Gel. AAPS Pharm. Sci. Tech..

[74] Anaya-Castro M.A., Ayala-Zavala J.F., Muñoz-Castellanos L., Hernández-Ochoa L., Peydecastaing J., Durrieu V. (2017). β-Cyclodextrin inclusion complexes containing clove (*Eugenia caryophyllata*) and Mexican oregano (*Lippia berlandieri*) essential oils: Preparation, physicochemical and antimicrobial characterization. Food Packag. Shelf Life.

[75] Hogenbom J., Jones A., Wang H.V., Pickett L.J., Faraone N. (2021). Synthesis and characterization of β-cyclodextrin-essential oil inclusion complexes for tick repellent development. Polymers.

[76] Hu Y., Qiu C., Qin Y., Xu X., Fan L., Wang J., Jin Z. (2021). Cyclodextrin–phytochemical inclusion complexes: Promising food materials with targeted nutrition and functionality. Trends Food Sci. Technol..

[77] Ferro D.N., Logan J.A., Voss R.H., Elkinton J.S. (1985). Colorado potato beetle (Coleoptera: Chrysomelidae) temperature-dependent growth and feeding rates. Environ. Entomol..

[78] Sladan S., Miroslav K., Ivan S., Snezana J., Petar K., Goran T., Jevdovic R. (2012). Resistance of Colorado potato beetle (Coleoptera: Chrysomelidae) to neonicotinoids, pyrethroids and nereistoxins in Serbia. Rom. Biotechnol. Lett..

[79] Maharijaya A., Vosman B. (2015). Managing the Colorado potato beetle; the need for resistance breeding. Euphytica.

[80] Scott I.M., Tolman J.H., MacArthur D.C. (2015). Insecticide resistance and cross-resistance development in Colorado potato beetle *Leptinotarsa decemlineata* Say (Coleoptera: Chrysomelidae) populations in Canada 2008–2011. Pest Manag. Sci..

[81] Cingel A., Savić J., Lazarević J., Ćosić T., Raspor M., Smigocki A., Ninković S. (2016). Extraordinary adaptive plasticity of Colorado Potato Beetle: “Ten-striped Spearman” in the era of biotechnological warfare. Int. J. Mol. Sci..

[82] Arthropod Pesticide Resistance Database (APRD) Leptinotarsa decemlineata-Shown Resistance to Active Ingredient(s). https://www.pesticideresistance.org/display.php?page=species&arId=141.

[83] IRAC 2023 Insecticide Resistance Action Committee. https://www.irac-online.org.

[84] Pour S.A., Shahriari M., Zibaee A., Mojarab-Mahboubkar M., Sahebzadeh N., Hoda H. (2022). Toxicity, antifeedant and physiological effects of trans-anethole against *Hyphantria cunea* Drury (Lep: Arctiidae). Pestic. Biochem. Physiol..

[85] Hummelbrunner L.A., Isman M.B. (2001). Acute, sublethal, antifeedant, and synergistic effects of monoterpenoid essential oil compounds on the tobacco cutworm, *Spodoptera litura* (Lep., Noctuidae). J. Agric. Food Chem..

[86] Shahriari M., Sahebzadeh N., Sarabandi M., Zibaee A. (2016). Oral Toxicity of Thymol, α-Pinene, Diallyl Disulfide and Trans-Anethole, and Their Binary Mixtures against *Tribolium castaneum* Herbst Larvae (Coleoptera: Tenebrionidae). Jordan J. Biol. Sci..

[87] Michaud D., Nguyen-Quoc B., Yelle S. (1993). Selective inhibition of Colorado potato beetle cathepsin H by oryzacystatins I and II. FEBS Lett..

[88] Cingel A., Savić J., Ćosić T., Zdravković-Korać S., Momčilović I., Smigocki A., Ninković S. (2014). Pyramiding rice cystatin OCI and OCII genes in transgenic potato (*Solanum tuberosum* L.) for resistance to Colorado potato beetle (*Leptinotarsa decemlineata* Say). Euphytica.

[89] Huang J.H., Jing X., Douglas A.E. (2015). The multi-tasking gut epithelium of insects. Insect Biochem. Mol. Biol..

[90] Rivard D., Cloutier C., Michaud D. (2004). Colorado potato beetles show differential digestive compensatory responses to host plants expressing distinct sets of defense proteins. Arch. Insect Biochem. Physiol..

[91] Hsiao T.H., Fraenkel G. (1968). The influence of nutrient chemicals on the feeding behavior of the Colorado potato beetle, *Leptinotarsa decemlineata* (Coleoptera: Chrysomelidae). Ann. Entomol. Soc. Am..

[92] Kivelä M., Friberg M., Wiklund C., Leimar O., Gotthard K. (2016). Towards a mechanistic understanding of insect life history evolution: Oxygen dependent induction of moulting explains moulting sizes. Biol. J. Linn. Soc..

[93] Furlong M.J., Groden E. (2003). Starvation induced stress and the susceptibility of the Colorado potato beetle, *Leptinotarsa decemlineata*, to infection by *Beauveria bassiana*. J. Invertebr. Pathol..

