# Supplementary material for: Encapsulation of Fennel and Basil Essential Oils in β-Cyclodextrin for Novel Biopesticide Formulation
Source: Biomolecules. 2024 Mar 14;14(3):353. doi: 10.3390/biom14030353 (PMC10968348; doi:10.3390/biom14030353)
Supplement: Supplementary file 1 [file biomolecules-14-00353-s001.zip › biomolecules-2890112-supplementary.pdf]

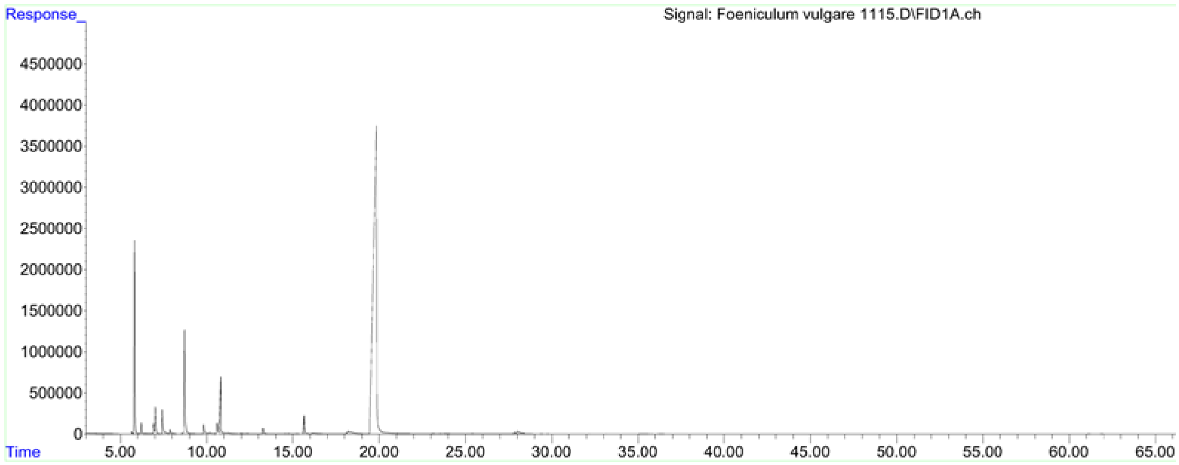

Figure S1: Chromatogram of GC/MS analysis of fennel (*Foeniculum vulgare* Mill.) essential oil

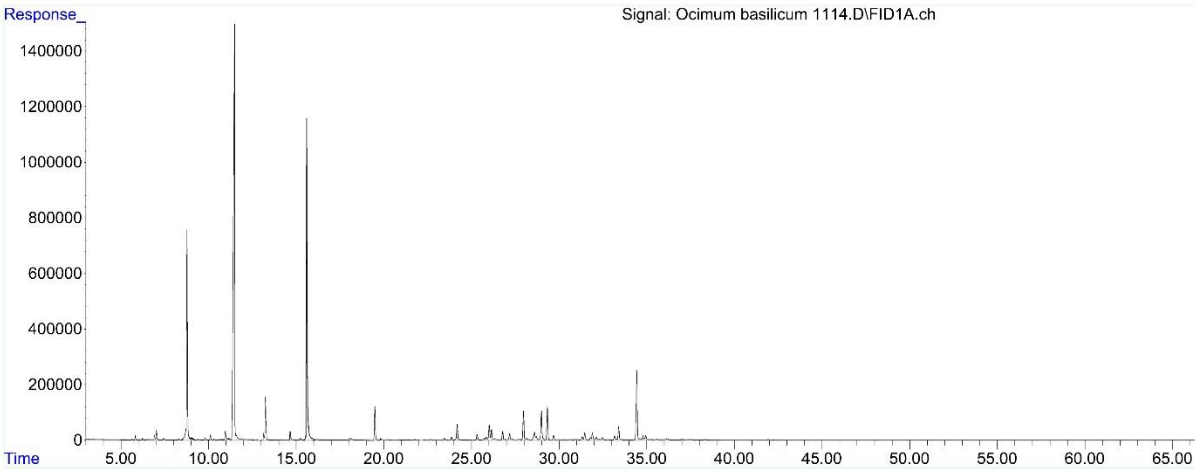

Figure S2: Chromatogram of GC/MS analysis of basil (*Ocimum basilicum* L.) essential oil
